# Multiple Splenic Artery Aneurysms: A Case Report and Review of the Literature

**DOI:** 10.3389/fsurg.2021.763890

**Published:** 2022-01-05

**Authors:** Wen Chun Chen, Tie hao Wang, Ding Yuan, Ji Chun Zhao

**Affiliations:** West China Hospital, Sichuan University, Chengdu, China

**Keywords:** splenic artery aneurysms, splenectomy, aneurysmectomy, leukopenia, thrombocytopenia, endovascular embolization

## Abstract

**Background:** Multiple splenic artery aneurysms (MSAAs) are rare and there are few reports about their treatment. We herein present a rare case of MSAAs treated with splenectomy combined with endovascular embolization.

**Methods:** A 51-year-old female patient was incidentally diagnosed with MSAAs. Splenectomy combined with endovascular embolization was the chosen treatment.

**Outcomes:** The patient recovered uneventfully and was discharged from the hospital 5 days after splenectomy. The patient has been doing well during the 27-months of follow-up.

**Conclusion:** Combined with the experience of the previous literature, we think splenectomy combined with endovascular embolization is a safe, reliable and minimally invasive treatment for some selected multiple SAAs, depending on several patient parameters, such as the age, sex, aneurysm dimension, aneurysm location, complications, and severity of the clinical findings.

## Introduction

Splenic artery aneurysm (SAA) is the most common visceral aneurysm. It comprises about 60% of all visceral aneurysm cases and occurs predominantly in multiparous women and portal hypertension patients (1–3). The main risk factors for true SAAs are hypertension, atherosclerosis, liver cirrhosis, portal hypertension (PHT), liver transplantation, women, pregnancy and multiple pregnancies, with pregnancy and PHT being the most important risk factors (1, 2). The incidence of SAA was reportedly 7–50% in patients diagnosed as having cirrhosis and portal hypertension, and the incidence of PHT was reportedly 50% in SAA patients (1–5). Portal hypertension with SAA is common, whereas portal hypertension with multiple splenic artery aneurysms (MSAAs) is rarely. According to previous literatures, true MSAAs have an estimated prevalence rate of 0.02–0.1% (6). Despite the rarity of MSAAs, they are clinically important because their possible rupture may be catastrophic. Available methods for treatment of SAAs include endovascular, laparoscopic, and open surgery. However, the treatment of MSAAs is challenging for vascular surgeons. Herein we present a rare case of MSAAs treated with splenectomy combined with endovascular embolization and review the relevant literature.

## Case Report

**Figure 1:**
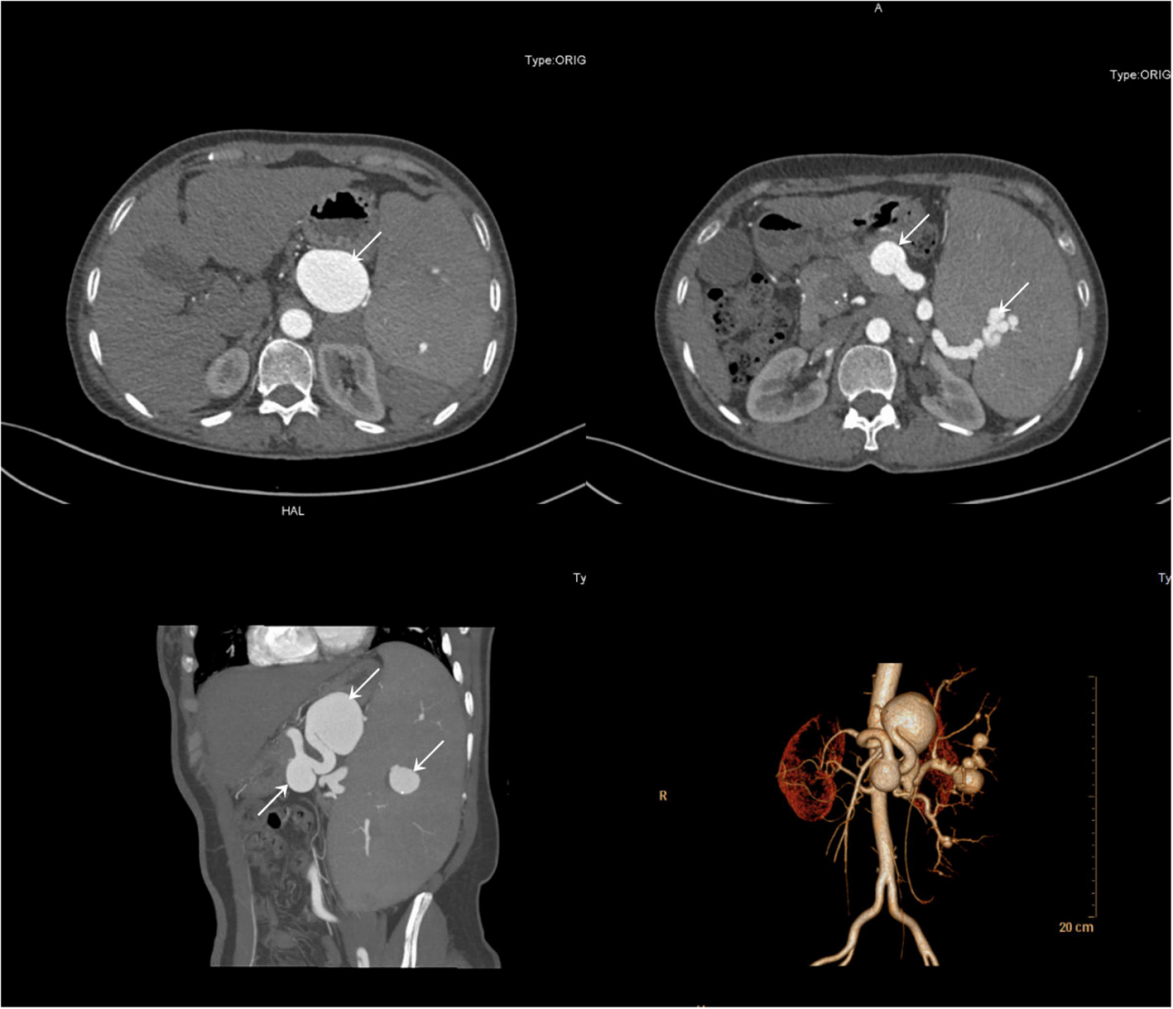
Computerized tomography angiography demonstrated multiple aneurysms of the splenic artery.

A 51-year-old female patient with a negative abdominal physical examination was admitted to our hospital for multiple splenic artery aneurysms incidentally detected because of her presentation of occasional pain in her left ribs. Her past medical history showed that she was previously diagnosed as having chronic hepatitis B. She did not have any history of a genetically inherited disease and had never undergone a surgery. She had a history of two pregnancies. Her initial lab test results showed a hemoglobin level of 117 g/L, red blood cell count of 3.64 × 10^12^/L, white blood cell count of 1.82 × 10^9^/L, and platelet count of 20 × 10^9^/L, whereas all other laboratory test results were normal. Subsequently, a computed tomographic angiography showed multiple aneurysms of the splenic artery, and the largest aneurysm was 4.6×3.5 cm (Figure 1). She was diagnosed with multiple splenic artery aneurysms, chronic hepatitis B, compensatory stage of liver cirrhosis, splenomegaly with hypersplenism, severe thrombocytopenia and portal hypertension. Considering the diameter of the aneurysms, the morphology of the aneurysms and splenomegaly with hypersplenism the treatment of aneurysmectomy plus splenectomy was obviously indicated. The patient has consented to the publication of the case details and images.

**Figure 2:**
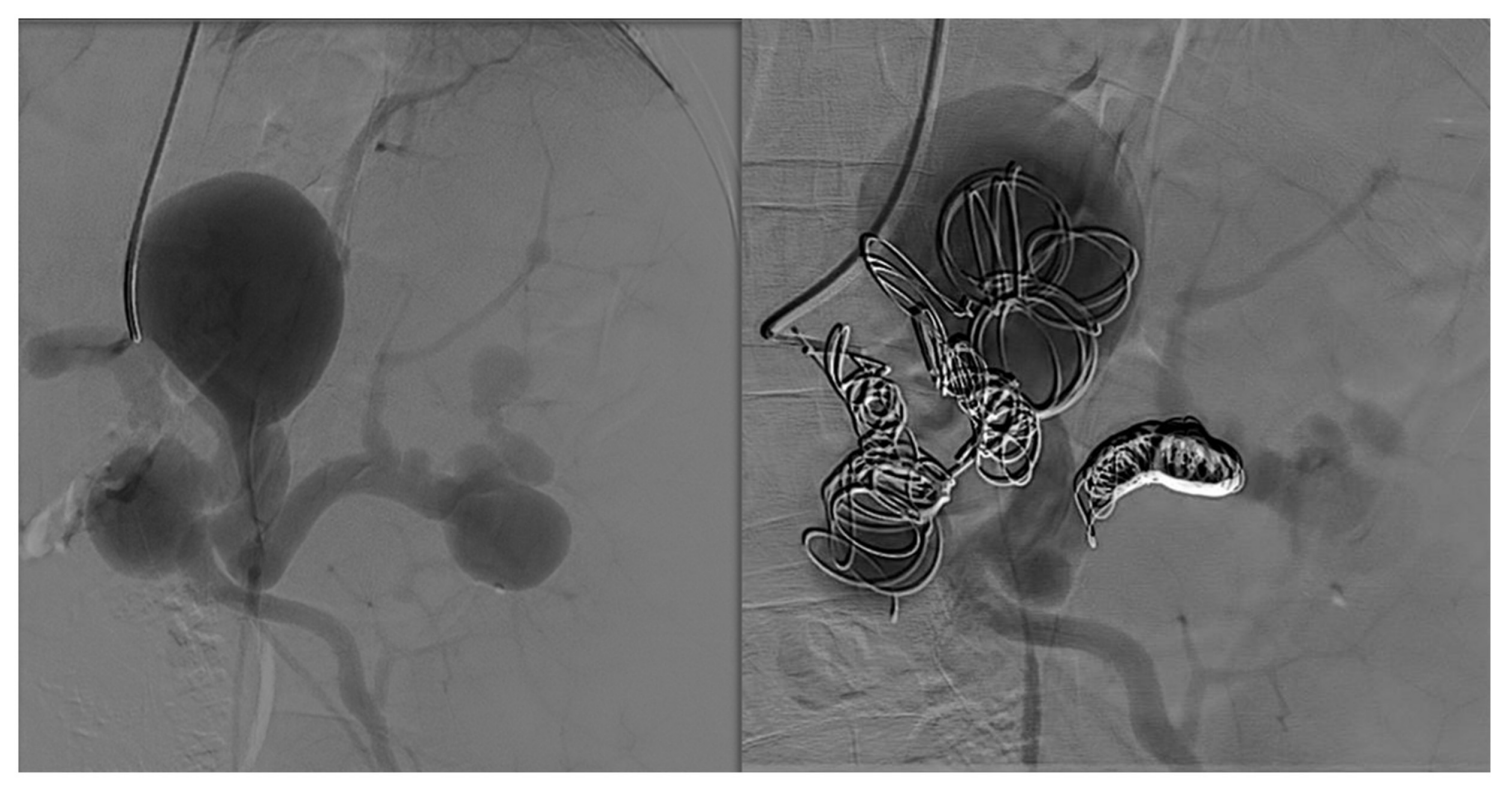
Selective angiography showed that the blood flow to the spleen was significantly reduced.

**Figure 3:**
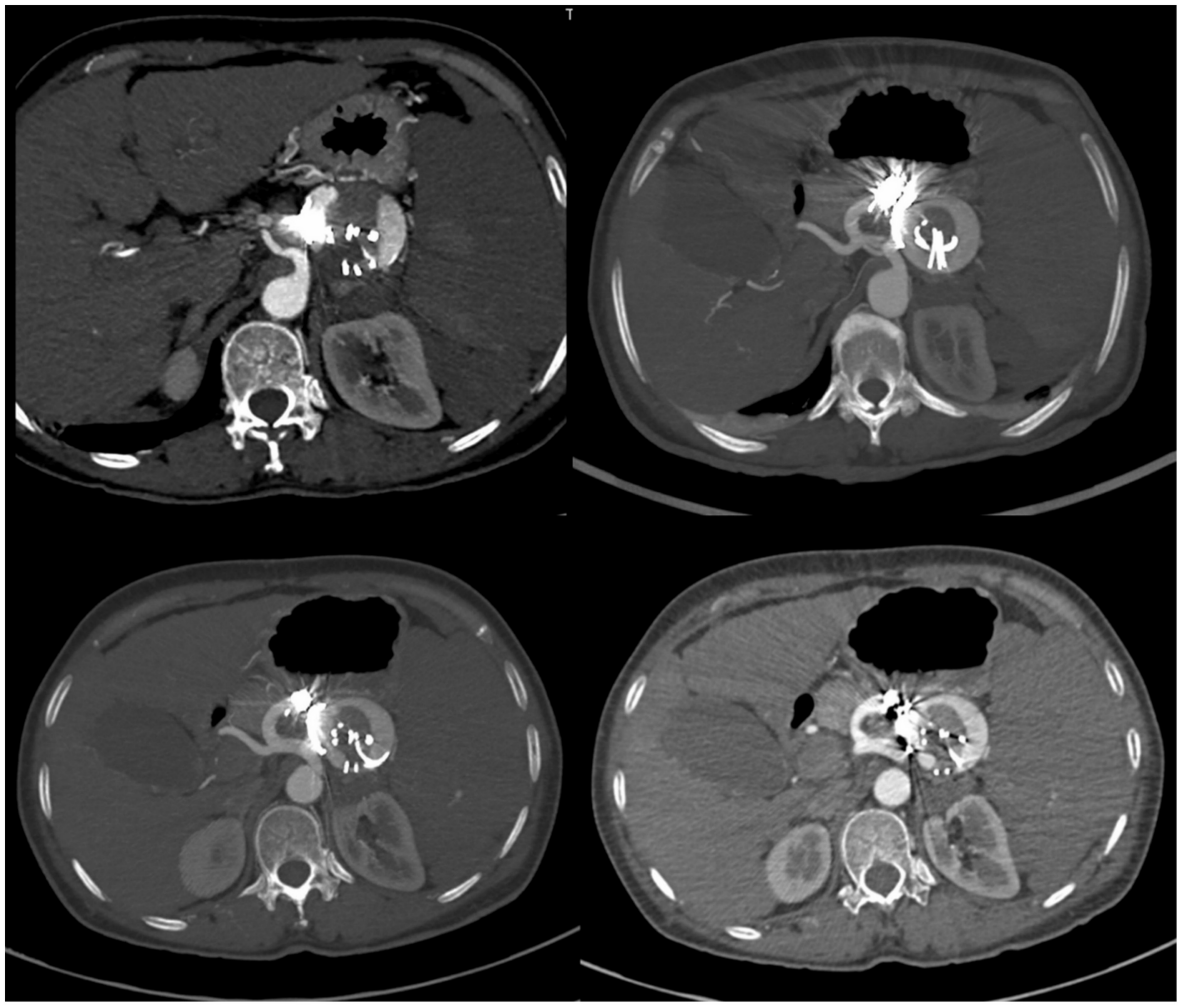
CT-scan of the abdomen showed signs of blood flow into the embolized splenic aneurysm sac.

First, we performed endovascular embolization and subsequently monitored the patient's leukocyte and platelet counts. The selective splenic artery angiography confirmed the multiple aneurysms along splenic artery (Figure 1). Then, two of the larger aneurysms were selected for spring coil partial embolization, while the splenic hilum aneurysms and intra-splenic aneurysms were not treated. Meanwhile we also embolized the outflow tract of the largest aneurysm. The patient's postembolization angiogram showed that the blood flow in the aneurysmal sac had significantly reduced and slowed down (Figure 2). After-embolization, the patient also did not experience any complication, such as bowel ischemia, pain, or fever. On the 4th day after endovascular embolization, her CT scan showed thrombosis in the aneurysm sac (Figure 3). One week after endovascular embolization, her white blood cell count was 3.74 × 10^9^/L and her platelet count was 72 × 10^9^/L. Subsequently, we performed aneurysmectomy plus splenectomy without any blood transfusion during the process. The patient was not transferred to the intensive care unit. The patient's platelet count and white blood cell count normalized at 2 and 3 days after surgery, respectively. She recovered uneventfully and was discharged from the hospital 5 days after surgery. During her 6-months of follow-up, her platelet count remained normal and no adverse events occurred.

## Discussion

SAAs are usually asymptomatic and diagnosed incidentally. Currently, the guidelines recommend treating non-ruptured splenic artery true aneurysms >3 cm, with a demonstrable increase in size, or with associated symptoms in patients of acceptable risk because of the risk of rupture (1 C)(7). MSAAs is rarely and the treatment was challenging. To the best of our knowledge, there are only 36cases previously reported in 25 English medical literatures to the date of writing in a search of PubMed, Google Scholar, and Google databases (Table 1).

**Table 1:** Clinical and treatment features of the reported cases of multiple splenic artery aneurysms.

**References**	**#**	**Age(years)** **and sex**	**Possible etiology**	**Symptoms**	**Rupture**	**MD/** **(mm)**	**Location**	**Intrasplenic/** **hilum SAA**	** Treatment**	**Outcome/** **Complication**
Matter, (8)	12	NA	Liver cirrhosis PHT Hypertension Atherosclerosis Trauma	2 cases: Cardiovascular collapse with acute abdominal pain 9 cases: symptomless	Yes: 2 cases No: 10 cases	50	Distal third of SA:8, middle third of SA:4	NA	Splenectomy with splenic artery ligation:5, close follow-up: 7	Lost follow-up: 1 died from ruptured SAAs: 2 survival: 9 No complication
Kitamura, (9)	1	64y, F	Connective tissue abnormalities	Left upper quadrant pain	Yes	31	Distal third of SA	Yes	Splenectomy with aneurysmectomy	Survival/No
Cho, (10)	1	54y, M	Trauma	Abdominal discomfort	No	45	Intrasplenic	Yes	Splenectomy	Survival/No
Juszkat, (11)	1	60y, F	NA	Vague upper abdominal pain	No	25	Distal third of SA	Yes	Splenectomy with aneurysmectomy and coil embolization	Survive/No
Woo, (12)	1	20y, M	Unknown	Flank pain	No	16	Distal third of SA	No	Splenectomy with splenic artery ligation	Survival/No
Ohmoto, (13)	1	59y, F	Liver cirrhosis PHT	Symptomless	No	24	Distal third of SA	No	Coil embolization	Survival/No
Williamson, (14)	1	71y, F	Liver cirrhosis PHT	Left upper quadrant pain and pulsating sensation	No	NA	NA	NA	Splenectomy with splenic artery ligation	Survival/No
Watada, (15)	1	64y, M	FD	Right lower abdominal pain	No	40	Distal third of SA	Yes	Splenectomy with splenic artery ligation	Survival/No
Zubaidi, (16)	1	42y, F	Multiple pregnancies	Left-sided abdominal pain lightheadedness vomiting hematemesis	Yes	45	Distal third of SA	Yes	Splenectomy with splenic artery ligation	Survival/No
Al-abbal, (4)	1	70y, M	NA	Left upper quadrant pain	No	40	Proximal two- third of SA	No	Coil embolization	Survival/No
Phillips, (17)	1	16y, M	Liver cirrhosis PHT	Symptomless	No	43	Distal third of SA	Yes	Close follow-up	Survival/No
Wang, (18)	1	25y, F	NA	Left upper quadrant abdominal pain, fever duration	No	35	Distal two- third of SA	Yes	Splenectomy with splenic artery ligation	Survival/No
Manjunatha, (19)	1	16y, F	PHT	Vague abdominal pain hematemesis abdominal mass	No	18	Distal third of SA	Yes	Splenectomy, aneurysmectomy and splenorenal shunt	Survival/No
Yakubovitch, 2013 (20)	1	32y, M	PHT	Acute onset of epigastric pain	Yes	25	Distal two- third of SA	Yes	Splenectomy with aneurysmectomy and coil embolization	Survival/No
Aroor, (21)	1	39y, F	FD	Abdominal discomfort duration	No	58	Distal third of SA	Yes	NA	Survival/No
Honda, (22)	1	74y, F	Pneumococcal pneumonia infection	Acute right upper quadrant pain	No	35	Intrasplenic	Yes	Coil embolization respectively	Survival/No
Beksac, (6)	1	23y, F	PHT	Symptomless	No	70	All segments	Yes	Aneurysmectomy with splenectomy	Survival/No
Bizueto, (23)	1	66y, F	NA	Occasional abdominal pain	No	90	Distal third of SA	No	Aneurysmectomy with revascularization	Survival/No
Termos, (24)	1	54y, F	Unknow	Left sided abdominal pain	No	25	Distal third of SA	No	Aneurysmectomy with splenectomy	Survival/No
Bagga, (25)	1	40y, F	Liver cirrhosis PHT	NA	No	20	Distal two- third of SA	Yes	Close follow-up	Survival/No
Niu, (26)	1	57y, F	Liver cirrhosis PHT	Symptomless	No	27	Distal two- third of SA	Yes	Coil embolization	Survival/No
Rehman, (27)	1	22y, F	PHT	Left upper abdominal pain	No	100	Distal third of SA	Yes	Glue and coils embolization	Survival/No
Sakamoto, (28)	1	36y, M	FD DLC	Symptomless	No	110	Distal two- third of SA	Yes	Splenectomy with distal pancreatectomy	Survival/No
Kawachi, (29)	1	51y, F	IPHT	NA	No	20	Distal third of SA	Yes	Living donor liver transplantation and splenectomy	Survival/No
Selim, (30)	1	39y, F	Trauma	NA	No	15	Distal third of SA	Yes	Coil embolization	Survival/No
Stoelting, (31)	1	58y, F	Multiple pregnancies	Nausea, vomiting early satiety weight loss	No	60	Distal two- third of SA	Yes	Partial embolization and diagnostic splenectomy	Survival/No

*MD, maximum diameter in multiple splenic artery aneurysms; NA, not available; FD, Fibromuscular dysplasia; PHT, portal hypertension; IPHT, Idiopathic portal hypertension; DLC, decompensated liver cirrhosis. # means number of patient*.

Currently, several treatment methods exist for treating SAA, including endovascular, laparoscopic, and open surgery. In recent years, endovascular therapy has been favored for being minimally invasive, offering rapid postoperative recovery, and providing a high comfort level to patients. Several endovascular treatment methods for SAA, mainly include coil embolization, placement of covered stents, plug deployment, gluing, and injection of endoluminal thrombin, polyvinyl alcohol, particles, or gel foam (1, 2, 32, 33). Endovascular therapy is preferred in cases involving surgical technical difficulty and in patients with high risk of open operative. In addition, this option is considered for lesions located in the artery proximal and ruptured aneurysm (34). Endovascular treatment options, such as transcatheter embolization, stents graft, plug deployment and injection of endoluminal thrombin, polyvinyl alcohol, particles, or gel foam, for SAAs depend on the aneurysm's dimension, location and anatomical parameters. However, the application of this technique is limited by intrasplenic MSAAs, infected (mycotic) SAA, tortuous arteries, decreased artery dimensions, and the location of the lesion.In addition, giant aneurysm was not suitable for endovascular treatment, as the aneurysm may involve the intestine, pancreas, and other neighboring organs. Owing to the increasing frequency of endovascular treatment, its main complications, such as coil and stent migration, splenic and intestinal infarction, hemorrhage and aneurysm rupture, fever, and splenic abscess and recanalization, have begun to attract the attention of clinicians (35–38).

Despite rapid advances in minimally invasive surgery, open abdominal surgery remains the gold standard for treatment. Open/laparoscopic surgery aneurysmectomy with/without splenectomy is still suitable for patients with distally located, multiple SAAs, giant SAA, intrasplenic SAA, infectious SAA and elongated and tortuous SAA (1, 2, 39, 40). In addition, this method is also suitable for patients with failure of endovascular treatment, complications with endovascular treatment (e.g., splenic infarction and graft displacement.), severe splenic infarction, splenic abscess and abdominal dense adhesion (1, 2, 33, 40, 41). Surgical treatment often involves the spleen, pancreas and other adjacent organs, and distal pancreatectomy is necessary. This depends on the dimensions of the lesion, coexisting morbidities (pancreatitis, cirrhosis, or portal hypertension), and the experience of the team (1, 2). However, the mortality and morbidity of open/ laparoscopic surgery are higher than those of endovascular treatment. Moreover, compared with endovascular surgery, open/laparoscopic surgery has the disadvantages of being more invasive, offering slower postoperative recovery and causing patients greater inconvenience. Laparoscopic surgery can be the optimal treatment and is minimally invasive, particularly when compared with open surgery, specifically in early pregnancy with smaller lesions. However, it is not applicable for giant aneurysm and having dense adhesion with surrounding tissues. Laparoscopic revascularization was not recommended. Extensive experience in endoscopic surgery was a prerequisite. There was no laparoscopic surgery in our literature review.

Our case, was of a patient having MSAAs with a giant sac, an intrasplenic aneurysm and a splenic hilum aneurysm. Considering the risk of splenic infarction, vascular recanalization and intrasplenic aneurysm rupture after endovascular embolization or stent graft, open abdominal surgery was initially planned. However, a patient with severe thrombocytopenia and leukopenia is a bad fit for open surgery. The prognosis of such patients may be poor when open surgery is abruptly performed. Partial embolization of splenic vessels is reportedly used to treat hypersplenism of thrombocytopenia, and partial splenic embolization is an effective method for improving the platelet count (2, 42–45). Embolization is primarily performed in the inflow and outflow tracts of aneurysms, and it is not necessary to completely embolize the aneurysm sac. After comprehensive consideration, endovascular partial embolization aneurysm was performed in the first step to relieve the patient's thrombocytopenia and leukopenia. Then aneurysmectomy with splenectomy was performed in the second step. Considering the patient had a giant aneurysm sac, intrasplenic aneurysm, splenic hilum aneurysm, and multiple aneurysms, we finally chose open abdominal aneurysmectomy with splenectomy and the patient achieved good results.

There is not a single treatment method suitable for all splenic aneurysms. A combination of several treatment techniques may be necessary for some cases, particularly for giant SAAs or patients with comorbid conditions. Endovascular therapy, laparoscopic surgery and open surgery options should be chosen after careful consideration of the patient's condition, which depends on several patient parameters, such as the age, sex, aneurysm dimension, aneurysm location, complications, and severity of the clinical findings. At the same time, we should also pay attention to regular monitoring, particularly after endovascular treatment.

## Conclusions

Endovascular treatment, laparoscopic surgery, and open surgery are important methods of treating SAAs. The preferred treatment of an individual patient and aneurysm must be carefully based on the particular anatomy and any associated clinical conditions as well as the underlying condition of the patient. For patients with multiple SAAs, particularly SAAs at the hilar or intrasplenic locations, and for those with more severe comorbidities, endovascular embolization combined with open surgery may be a good treatment choice.

## Data Availability Statement

The original contributions presented in the study are included in the article/supplementary material, further inquiries can be directed to the corresponding author/s.

## Author Contributions

WC was the first authors and wrote the manuscript and was assistant in surgery. TW and DY was involved in editing the manuscript and assistant in surgery. JZ was chief operating surgeon. All authors contributed to the article and approved the submitted version.

## Conflict of Interest

The authors declare that the research was conducted in the absence of any commercial or financial relationships that could be construed as a potential conflict of interest.

## Publisher's Note

All claims expressed in this article are solely those of the authors and do not necessarily represent those of their affiliated organizations, or those of the publisher, the editors and the reviewers. Any product that may be evaluated in this article, or claim that may be made by its manufacturer, is not guaranteed or endorsed by the publisher.
